# Atlas of Pathological Histology

**Published:** 1853-08

**Authors:** 


*Atlas of Pathological Histology.* By Dr. Gottlieb Gluge, Professor of Physiology and Pathological Anatomy in the Univercity of Bruxelles, &c. Translated from the German by Joseph Leidy, M.D., Pathologist to St. Joseph's Hospital, &c.; with three hundred and twenty figures, plain and colored, on twelve copper plate engravings. Philadelphia: Blanchard & Lea, 1853.

The introduction contains tables of the weight and magnitude of the organs of the human body, both in their normal and abnormal condition. The first section of the work treats of the development of the elements of tissues. Section second treats of the elements of the tissues, combined inperfect or imperfect tissues, and arranged according to the processof disease. Section third is on the formation of the blastema. Section fourth, the histological metamorphosis of the blood. Section fifth, pyæma. Section sixth, gangrene. Section seventh, which closes the body of the work, consists of observations on histology.

We cannot speak too highly of the intrinsic value of this work or is mechanical execution by those unrivaled publishers Blanchard & Lea.

The profession are under obligation to Dr. Leidy for this translation, so indispensable to every medical man who wishes to keep pace with the progress of physiological and pathological science.

